# COVID-19 mortality risk among women with ovarian cancer: a matched case-control study

**DOI:** 10.1590/1414-431X2026e15084

**Published:** 2026-04-27

**Authors:** K.P.I. Peruchi, V.A.F. Bastos, L.A. Teixeira, F.F. Pimentel, F.J. Candido dos Reis

**Affiliations:** 1 Departamento de Ginecologia e Obstetrícia, Faculdade de Medicina de Ribeirão Preto, Universidade de São Paulo, Ribeirão Preto, SP, Brasil; 2 Laboratório de Bioquímica, Instituto de Biotecnologia, Universidade Federal de Uberlândia, Uberlândia, MG, Brasil

**Keywords:** COVID-19, Ovarian neoplasms, Matched case-control study, Mortality, COVID-19 vaccines

## Abstract

Women with ovarian cancer may be at increased risk of severe COVID-19 outcomes. This study aimed to compare mortality and clinical outcomes between women with severe COVID-19, with and without a history of ovarian cancer. We conducted a matched case-control study using national surveillance data. Cases included women with severe COVID-19 and a history of ovarian cancer; controls were women with severe COVID-19 without such history. Matching was done at a 1:4 ratio using age, comorbidities, vaccination status, diagnosis date, and region. The primary outcome was COVID-19-related mortality. A total of 474 ovarian cancer cases and 1,896 controls were included. Mortality was significantly higher in women with ovarian cancer (54.9 *vs* 32.7%, P<0.001). Multivariate analysis showed that ovarian cancer increased the risk of death (OR: 2.76, 95%CI: 2.22-3.43). Age also influenced mortality: OR 2.57 (95%CI: 2.11-3.13) for women aged 65-84, and OR 3.86 (95%CI: 2.52-5.97) for those 85 and older. Vaccination provided protection: complete vaccination (OR: 0.67, 95%CI: 0.51-0.88) and complete vaccination plus booster (OR: 0.35, 95%CI: 0.27-0.47). Women with ovarian cancer had a significantly higher risk of death from severe COVID-19. Vaccination, particularly with a booster, was associated with 65% reduced mortality.

## Introduction

Ovarian cancer is one of the most lethal gynecologic malignancies, affecting 324,398 women globally in 2022 and accounting for 206,839 deaths (1). Over 60% of cases are diagnosed at an advanced stage (2), posing significant management challenges and requiring a well-coordinated, multidisciplinary approach. Patient outcomes improve considerably when treatment is delivered in centralized, specialized settings (3). Standard care typically involves aggressive upfront surgery followed by chemotherapy and maintenance therapy, or neoadjuvant chemotherapy followed by surgical debulking and maintenance therapy (4). Despite these interventions, complete and functional recovery is not always achievable, and many survivors experience long-term disabilities that profoundly affect their life (5).

The COVID-19 pandemic created unprecedented challenges for healthcare systems worldwide. From the first case identified in Wuhan, China (6), to December of 2023, over 772 million individuals were infected, and nearly 7 million deaths were attributed to the disease (7). The pandemic severely disrupted routine healthcare services, including cancer care, leading to delayed diagnoses, interruptions in treatment, and heightened psychological stress among patients. The full extent of the pandemic's impact on women with ovarian cancer remains unclear, though outcomes likely depended on the healthcare system's capacity and priorities during the crisis (8,9). Furthermore, ovarian cancer itself, along with comorbidities associated with its treatment, may have influenced outcomes for patients infected with SARS-CoV-2.

This study aimed to compare the outcomes of women with COVID-19 severe acute respiratory syndrome (SARS) with and without a history of ovarian cancer.

## Material and Methods

### Setting

In Brazil, the Ministry of Health, through the Health Surveillance Secretariat, has monitored SARS since the influenza A (H1N1) pandemic in 2010. In 2020, COVID-19 surveillance was incorporated into this network. Severe COVID-19 was defined as an individual with flu-like symptoms who presents with one of the following: shortness of breath/respiratory distress, persistent pressure or pain in the chest, oxygen saturation below 95% in room air, or bluish coloration (cyanosis) of the lips or face. This definition is in alignment with the definition of severe COVID-19 for epidemiological purposes (10).

The hospitalized severe COVID-19 cases and pre-hospital deaths are recorded in the surveillance system, with raw data accessible on the Open Data SUS (Brazil’s public health system) platform at https://opendatasus.saude.gov.br/dataset/?q=srag. Data are collected by healthcare professionals and administrators across Brazil's health system, including hospitals, clinics, and laboratories. The Brazilian Ministry of Health manages the surveillance system through the Department of Informatics of the SUS (DATASUS), ensuring data integration and dissemination. Data quality is maintained through automated validation rules and manual reviews, but inconsistencies may arise due to variations in reporting practices. Publicly available datasets comply with Brazil's General Data Protection Law (LGPD), which restricts the access to sensitive patient information.

According to Brazilian ethical research norms, Resolution No. 674, of May 6, 2022, Chapter II, Article 2, Section XII, research that uses publicly accessible information, which is not subject to privacy, security, or access limitations, is exempt from submission to the Research Ethics Committee and National Counsel for Research Ethics (CEP/CONEP) system. The data used in this study are anonymous and do not allow for the direct or indirect identification of the individuals related to them. This study adhered to the principles of the Declaration of Helsinki, ensuring respect for participants, maintaining confidentiality, providing scientific and social value, and committing to transparency in publishing findings.

### Study design

This was a propensity score-matched case-control study. Participants were selected between March 2020 and December 2024. Each ovarian cancer case was matched to four controls using variables such as age at COVID-19 diagnosis, comorbidities (e.g., cardiovascular diseases, hematological disorders, liver disease, asthma, diabetes, neurological conditions, lung disease, immunosuppression, renal disease, and obesity), vaccination status, year and month of diagnosis, and treatment location. The nearest-neighbor matching method was applied based on a logistic regression-derived propensity score, ensuring well-balanced groups and minimizing bias.

### Participants

Cases included individuals with “ovarian cancer” listed in the “description of other comorbidities” field on the notification form, where COVID-19 was the cause of SARS, and outcome data were complete. Controls were individuals without reported ovarian cancer, with COVID-19 as the cause of SARS, and complete outcome data.

### Variables

The primary exposure variable in this study was history of ovarian cancer, categorized as “Case” or “Control”. Outcome variables included COVID-19 outcomes classified as “Recovery” or “Death”, and the need for ventilation support, categorized as “No”, “Non-invasive”, or “Invasive”. Age was calculated in years based on the date of the first symptom and the date of birth, and was grouped into three categories for multivariate analysis: “<64”, “65-84”, and “85+”. Vaccination status was classified as “Unvaccinated”, “Incomplete vaccination”, “Complete vaccination” (two doses of any vaccine or one dose of Janssen), and “Complete vaccination + booster”. The presence of comorbidities was categorized as “Yes” or “No”, including cardiovascular diseases, hematological disorders, liver disease, asthma, diabetes, neurological conditions, lung disease, immunosuppression, renal disease, and obesity. Additional covariates included the major Brazilian region of record notification, which was used to account for geographic variation in healthcare access and reporting practices. These variables were included in both matching and multivariate analyses to minimize bias and control for confounding factors.

Ovarian cancer diagnoses were identified from the open field ‘description of other comorbidities' and confirmed through manual verification by the research team. Only records in which ovarian cancer was explicitly reported by healthcare professionals were included. Entries suggesting possible confusion with uterine, cervical, or other gynecologic cancers were excluded after detailed review to ensure diagnostic accuracy.

### Study size

A systematic process determined the study size. Initially, 474 cases meeting the inclusion criteria were identified. Case-control ratios of 1:1, 1:2, 1:3, and 1:4, were tested for balance in risk factors associated with SARS outcomes. All ratios produced balanced groups, and a 1:4 ratio was chosen to maximize statistical power.

### Bias

To minimize bias, propensity score matching was used with a 1:4 case-control ratio, balancing covariates associated with poor COVID-19 outcomes. Authors manually verified open-field entries identifying ovarian cancer cases. Only patients with confirmed COVID-19 diagnoses were included, and those with missing outcomes or incongruent data were excluded. These measures enhanced the reliability of the findings.

### Statistical methods

Statistical analyses were performed using R version 4.4.1 in RStudio version 2024.04.2 Build 764 (USA). Data manipulation utilized libraries such as *dplyr*, *lubridate*, *data.table*, *MatchIt*, and *labelled*. Tabulation and visualization were conducted with *table1*, *MASS*, *ggplot2*, and *forcats*. Initial comparisons used chi-squared test or *t*-test, as appropriate. Multivariate logistic regression (binomial) was used to identify independent predictors of mortality. Stepwise Akaike Information Criterion (AIC) model selection was applied to refine the final multivariate model and isolate potential confounders.

## Results

**Figure 1:**
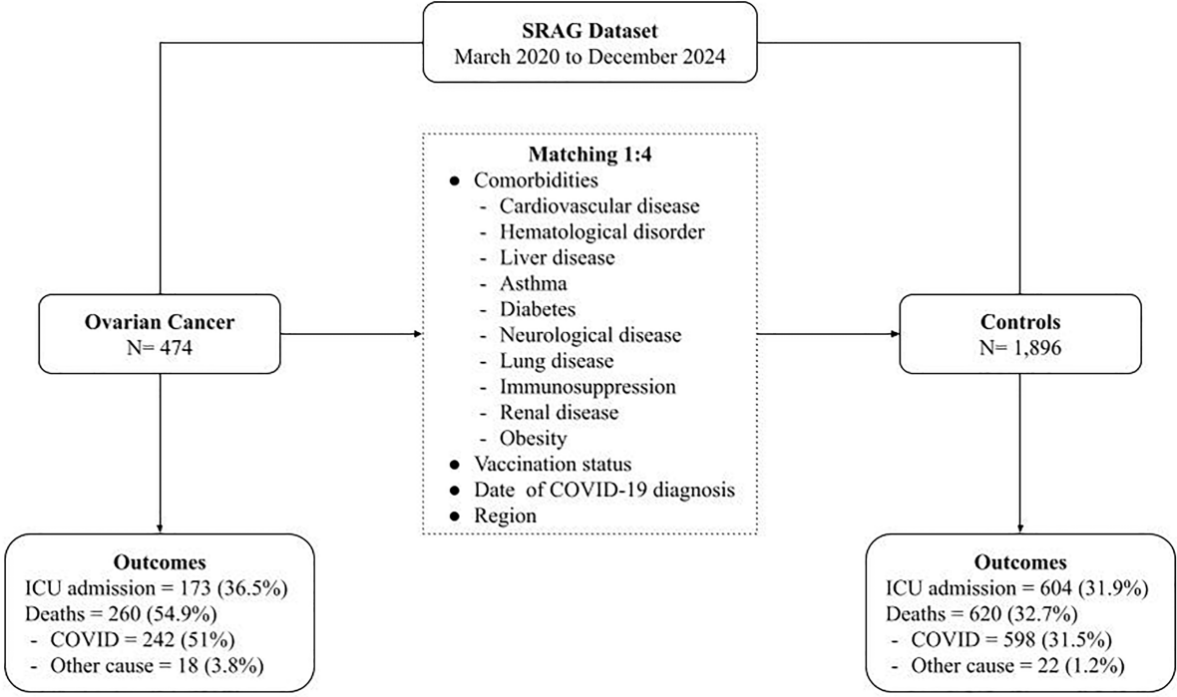
Flowchart of study selection, sample matching, and outcomes.

We identified 474 records of women with a history of ovarian cancer and severe COVID-19. These records were matched with 1,896 records of women with severe COVID-19 without a history of ovarian cancer, forming a total cohort of 2,370 participants. Figure 1 shows the flowchart of case selection, matching process, and main outcomes. The mean age of participants was 61.4 years (SD: 15.7). The prevalence of comorbidities, including cardiovascular disease (22.6%), diabetes (15.9%), and immunodeficiency (22.8%), was balanced between cases and controls. Regional distribution and vaccination status were also similar across groups, with over two-thirds of participants being unvaccinated. Table 1 provides a detailed overview of participants' characteristics.

**Table 1:** Characteristics of the participants.

	Control(n=1,896)	Ovarian cancer(n=474)	P	Total(n=2,370)
Age (mean (SD))	61.4 (15.8)	61.7 (15.3)	0.733	61.4 (15.7)
Cardiovascular disease	428 (22.6%)	107 (22.6%)	0.931	535 (22.6%)
Hematological disease	22 (1.2%)	7 (1.5%)	0.842	29 (1.2%)
Liver disease	26 (1.4%)	6 (1.3%)	0.982	32 (1.4%)
Asthma	24 (1.3%)	10 (2.1%)	0.382	34 (1.4%)
Diabetes	301 (15.9%)	76 (16.0%)	0.946	377 (15.9%)
Neurological disease	38 (2.0%)	11 (2.3%)	0.872	49 (2.1%)
Lung disease	52 (2.7%)	14 (3.0%)	0.755	66 (2.8%)
Immunodeficiency	433 (22.8%)	107 (22.6%)	0.903	540 (22.8%)
Kidney disease	45 (2.4%)	13 (2.7%)	0.897	58 (2.4%)
Obesity	94 (5.0%)	28 (5.9%)	0.704	122 (5.1%)
Region			0.731	
Central-West	211 (11.1%)	53 (11.2%)		264 (11.1%)
North	77 (4.1%)	17 (3.6%)		94 (4.0%)
Northeast	316 (16.7%)	79 (16.7%)		395 (16.7%)
South	319 (16.8%)	92 (19.4%)		411 (17.3%)
Southeast	973 (51.3%)	233 (49.2%)		1,206 (50.9%)
Year			0.807	
2020	605 (31.9%)	147 (31.0%)		752 (31.7%)
2021	739 (39.0%)	198 (41.8%)		937 (39.5%)
2022	431 (22.7%)	101 (21.3%)		532 (22.4%)
2023	89 (4.7%)	22 (4.6%)		111 (4.7%)
2024	32 (1.7%)	6 (1.3%)		38 (1.6%)
Vaccination Status			0.808	
Unvaccinated	1,273 (67.1%)	323 (68.1%)		1,596 (67.3%)
Incomplete	128 (6.8%)	28 (5.9%)		156 (6.6%)
Complete	239 (12.6%)	55 (11.6%)		294 (12.4%)
Complete + Boost	256 (13.5%)	68 (14.3%)		324 (13.7%)

Data are reported as mean and standard deviation or number and percent. P>0.05, *t*-test or chi-squared test.

Invasive ventilation was required more frequently in the ovarian cancer group (19.4%) compared to the control group (16.8%) (P=0.009). Non-invasive ventilation was also more common among ovarian cancer patients (49.4 *vs* 43.6%). A total of 54.9% of ovarian cancer patients died, a proportion significantly higher than the 32.7% in the control group (P<0.001).

**Figure 2:**
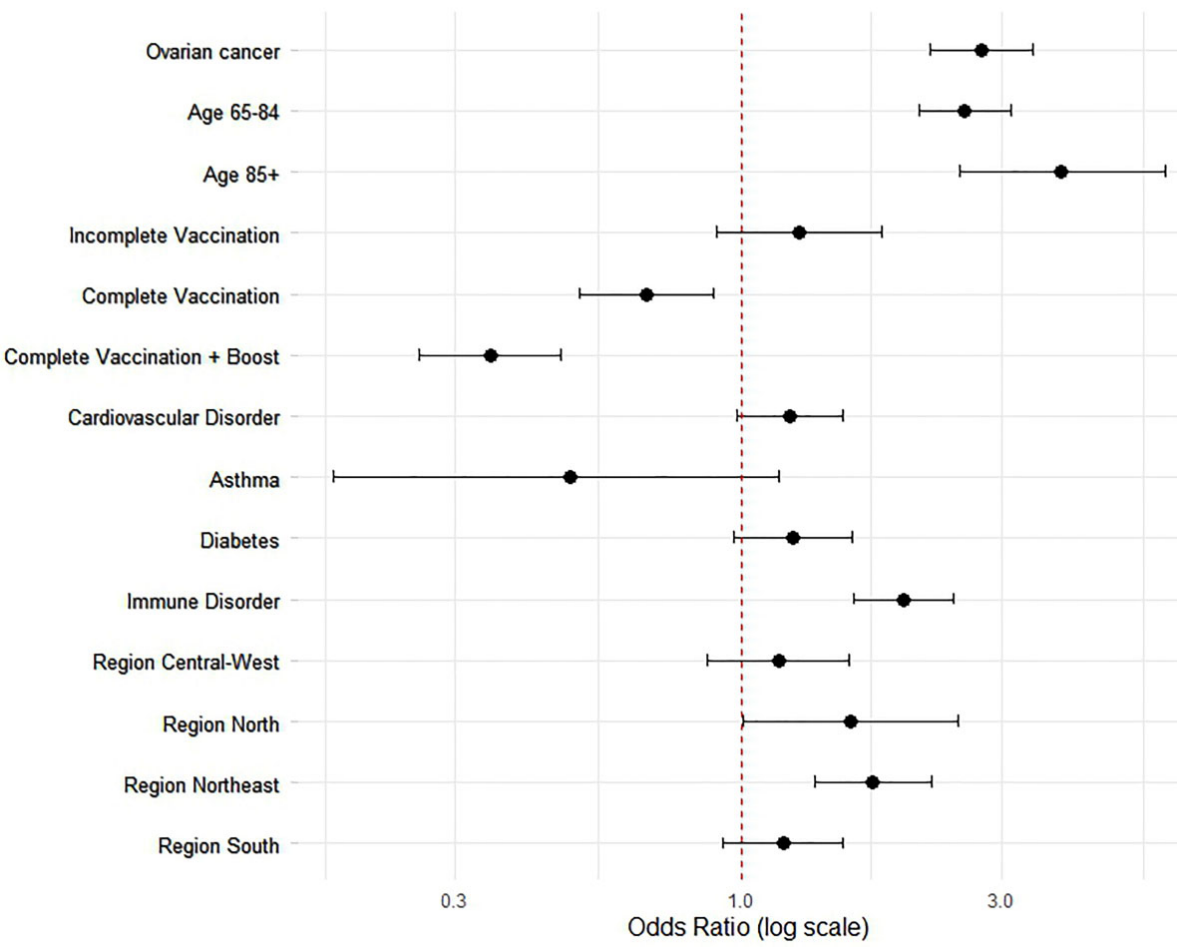
Multivariate logistic regression showing odds ratios and 95% confidence intervals for mortality risk factors in women with severe COVID-19.

Figure 2 illustrates the results of a multivariate analysis of risk factors for mortality in women with severe COVID-19. Ovarian cancer emerged as a strong predictor of increased mortality, with an odds ratio (OR) of 2.76 (95%CI: 2.22-3.43). Advanced age also significantly increased mortality risk, with ORs of 2.57 (95%CI: 2.11-3.13) for individuals aged 65-84 and 3.86 (95%CI: 2.52-5.97) for those aged 85 and older. Immune disorders were associated with a significantly higher risk of death (OR 1.99, 95%CI: 1.61-2.45).

Vaccination status showed a protective effect, with complete vaccination reducing the risk of mortality (OR: 0.67, 95%CI: 0.51-0.88) and booster doses conferring even stronger protection (OR: 0.35, 95%CI: 0.27-0.47). Significant regional disparities were observed, with higher mortality in the Northeast (OR 1.74, 95%CI: 1.36-2.23) and North (OR 1.59, 95%CI: 1.00-2.49) regions.

These findings underscore the substantial impact of ovarian cancer, advanced age, immune disorders, and geographic factors on COVID-19 mortality. Simultaneously, they highlight the critical protective role of vaccination and booster doses in mitigating these risks.

## Discussion

Our study demonstrated that women with severe COVID-19 and a history of ovarian cancer face a significantly higher risk of mortality compared to matched women with severe COVID-19 but without a history of ovarian cancer. These findings align with prior research indicating that cancer patients, particularly those with advanced malignancies, were at elevated risk of severe outcomes from COVID-19 mortality, especially before vaccines became available (11). Our findings are consistent with those reported in recent large-scale analyses. A recent systematic review and meta-analysis of 33 studies demonstrated that cancer was associated with a substantially higher risk of mortality compared to the general population (12), supporting our observation that women with ovarian cancer had significantly higher COVID-19 mortality even after adjusting for age, comorbidities, and vaccination status.

Cancer patients are especially susceptible to severe infections due to their immunocompromised state and the complexity of cancer treatment (13). During the COVID-19 pandemic, these vulnerabilities were magnified, leading to worse outcomes, such as increased hospitalization rates and higher mortality (14-17). Our findings highlight the disproportionate risks faced by ovarian cancer patients, who experienced significantly worse outcomes than the general population with COVID-19.

Most studies on ovarian cancer during the pandemic focused on disruptions in diagnosis and treatment rather than the increased risk of adverse outcomes. The pandemic caused a significant reduction in ovarian cancer diagnoses and surgical procedures across various cohorts (9,18-23). Factors contributing to these declines included healthcare system reorganization to prioritize COVID-19 care, resource reallocation, service interruptions, restricted healthcare access, and patients' fear of contracting COVID-19 at medical facilities. These challenges further delayed timely ovarian cancer management.

As the pandemic evolved, gynecologic oncology centers worldwide adapted their treatment strategies, transitioning from surgery to neoadjuvant chemotherapy, increasing the number of chemotherapy cycles, expanding the use of oral chemotherapy, and reducing intravenous chemotherapy (8,19,24-29). Together, these findings highlight the persistent excess risk of death from COVID-19 among patients with cancer, as demonstrated in multicenter cohorts and systematic reviews (12,15,16). The magnitude of this risk in our matched case-control study aligns with the broader literature and emphasizes the importance of targeted prevention strategies, including timely vaccination and early therapeutic interventions.

The strengths of this study include its robust matched case-control design and the use of comprehensive national data, which enhance the generalizability of the findings. The large sample size and detailed matching process controlled for potential confounders, providing more reliable estimates of the impact of ovarian cancer on COVID-19 outcomes. However, several limitations should be considered. The retrospective nature of the data introduces the possibility of reporting biases and unmeasured confounders, even with matching efforts. Excluding patients with missing data may have also introduced bias. Moreover, the lack of information on tumor histology, stage, and prior oncological treatments limits the analysis and interpretation of outcomes. Lastly, restricting the study to Brazilian patients may affect its generalizability to other populations.

The findings have important implications for clinical practice and healthcare policy. Given the elevated risk of severe COVID-19 outcomes in ovarian cancer patients, prioritizing these patients for preventive measures such as vaccination and early therapeutic interventions is critical. During future pandemics, healthcare systems should strive to maintain uninterrupted cancer care services to mitigate risks associated with treatment delays and interruptions.

Further research is essential to explore the long-term impacts of COVID-19 on cancer patients, including potential effects on cancer progression and survival. Studies examining various cancer types, the effectiveness of interventions in preventing severe outcomes, and the role of new therapeutic agents, early interventions, and vaccination strategies could provide critical insights for improving care in this population. Although the COVID-19 pandemic posed significant challenges to cancer care, advancements in vaccination, healthcare preparedness, and disease knowledge in the post-vaccination era suggest that a dual diagnosis of cancer and COVID-19 may now be associated with more favorable outcomes.

## Conclusion

In conclusion, our study underscores the significant risk of severe COVID-19 outcomes in women with ovarian cancer. These findings highlight the urgent need for tailored healthcare strategies to address the vulnerabilities of this population. Ensuring timely and effective care for ovarian cancer patients, even during a global healthcare crisis, remains paramount.

## Data Availability

The curated dataset and R analysis script can be obtained from the authors upon reasonable request.
